# Induction of tolerogenic dendritic cells by activated TGF-β/Akt/Smad2 signaling in RIG-I-deficient stemness-high human liver cancer cells

**DOI:** 10.1186/s12885-019-5670-9

**Published:** 2019-05-14

**Authors:** Ming Zhong, Cheng Zhong, Wen Cui, Guanghui Wang, Gongpu Zheng, Li Li, Jing Zhang, Rujing Ren, Huijei Gao, Tingting Wang, Xin Li, Jiantu Che, Eiichi Gohda

**Affiliations:** 1 Institute of Tumor Pharmacology, Jining Medical College, Xueyuan Road 669, Rizhao, 276826 China; 2 Division of Stem Cell Dynamics, Center for Stem Cell Biology and Regenerative Medicine, Institute of Medical Science, The University of Tokyo, Tokyo, Japan; 3 People’s Hospital of Rizhao, Rizhao, China; 4 S&V Biological Science and Technology Co., Ltd., Beijing, China; 5 Division of Pharmaceutical Sciences, Okayama University Graduate School of Medicine, Dentistry and Pharmaceutical Sciences, Okayama, Japan

**Keywords:** Tumor-infiltrating dendritic cell, Immune tolerance, Mixed lymphocyte reaction, Tumor microenvironment, Hepatocellular carcinoma

## Abstract

**Background:**

Dendritic cells (DCs) alter their role from being immunostimulatory to immunosuppressive at advanced stages of tumor progression, but the influence of cancer stem cells (CSCs) and their secreted factors on generation and phenotypic change of DCs is unknown. Retinoic acid-inducible gene I (RIG-I) plays a role in regulation of other cellular processes including leukemic stemness besides its antiviral function.

**Methods:**

Short hairpin RNA-mediated gene silencing was employed to generate stable RIG-I-knocked-down human hepatocellular carcinoma (HCC) cell lines. Expression levels of genes and proteins in spheres of those HCC cells were determined by quantitative real-time PCR and Western bot, respectively. Levels of secreted cytokines were measured by ELISA. The surface molecule expression levels of DCs were analyzed using flow cytometry. The ability of DCs to induce proliferation of T cells was assessed by a mixed lymphocyte reaction (MLR) assay.

**Results:**

RIG-I-knocked-down HCC cells showed upregulated expression of stem cell marker genes, enhanced secretion of factors suppressing in vitro generation of DCs into the conditioned medium (CM), and induction of a phenotype of tumor-infiltrating DCs (TIDCs) with low levels of DC markers in their tumors in nude mice. Those DCs and TIDCs showed reduced MLR, indicating RIG-I deficiency-induced immunotolerance. The RIG-I-deficient HCC cells secreted more TGF-β1 than did reference cells. The tumors formed after injection of RIG-I-deficient HCC cells had higher TGF-β1 contents than did tumors derived from control cells. DC generation and MLR suppressed by the CM of RIG-I-deficient HCC cells were restored by an anti-TGF-β1 antibody. TGF-β1-induced phosphorylation of Smad2 and Akt was enhanced in RIG-I-deficient HCC spheres, knockdown of *AKT* gene expression abolishing the augmentation of TGF-β1-induced Smad2 phosphorylation. Akt and p-Akt were co-immunoprecipitated with Smad2 in cytoplasmic proteins of RIG-I-deficient spheres but not in those of control spheres, the amounts of co-immunoprecipitated Akt and p-Akt being increased by TGF-β stimulation.

**Conclusions:**

Our results demonstrate that RIG-I deficiency in HCC cells induced their stemness, enhanced secretion and signaling of TGF-β1, tolerogenic TIDCs and less generation of DCs, and the results suggest involvement of TGF-β1 in those RIG-I deficiency-induced tolerogenic changes and involvement of CSCs in DC-mediated immunotolerance.

**Electronic supplementary material:**

The online version of this article (10.1186/s12885-019-5670-9) contains supplementary material, which is available to authorized users.

## Background

Dendritic cells (DCs) are antigen-presenting cells that find, capture, process and present antigens on the cell surface along with appropriate costimulation molecules such as the B7 family, tumor necrosis factor family and intracellular adhesion molecules. They activate and differentiate CD4^+^ and CD8^+^ T cells to develop an adaptive immune response. DCs are a pivotal immune component in the tumor microenvironment that consists of infiltrating immune cells, endothelial cells, extacellular matrix, and signaling molecules, and they affect tumor progression [1]. Recent studies have shown that DCs may alter their role from being immunostimulatory to immunosuppressive at advanced stages of tumor progression by being replaced with an immunosuppressive or regulatory DC phenotype and maintain immune tolerance, although the underlying mechanism is not fully understood [1–3].

Cancer stem cells (CSCs) represent a small subset of tumor cells that have the ability to self-renew and generate diverse cells that comprise the tumor bulk. Although CSCs and their factors secreted into the tumor microenvironment may affect the generation and phenotypic change of DCs, only a few studies on those activities of CSCs have been carried out. Grange et al. reported that renal CSCs impair the differentiation of DCs from monocytes by a mechanism involving human leukocyte antigen (HLA)-G [4]. A recent study by Li et al. has shown that retinoic acid-inducible gene-I (RIG-I), a pivotal cytoplasmic molecular pattern recognition receptor, is involved in the regulation of maintenance of leukemic stemness: RIG-I deficiency increases leukemic stemness [5]. This fact prompted us to investigate the relationship between stemness of cancer cells and the generation and phenotypic change of DCs.

RIG-I, originally identified as a gene induced by retinoic acid in a human promyelocytic leukemia cell line, has been documented as a pattern recognition receptor that detects cytoplasmic viral nucleic acids in virus-infected cells [6, 7]. Upon recognition of viral nucleic acids, RIG-I is activated from an inactive form via ATP hydrolysis-dependent conformational change and interacts with the mitochondrial adaptor molecule interferon (IFN)-β promoter stimulator-1 (IPS-1), leading to recruitment of TANK-binding kinase (TBK) 1, inducible IκB kinase (IKK-i), and IKKα/IKKβ/IKKγ. The corresponding transcriptional factors IFN regulatory factor (IRF)-3, IRF-7 and NF-κB are then activated, translocated into nucleus and induce transcription of type I IFN gene. Secreted IFN binds to the IFN receptor in an autocrine or paracrine manner and upregulates a variety of IFN-stimulated genes (ISGs) through Janus kinase (JAK)–signal transducers and activator of transcription (STAT) signaling pathways, thus inducing antiviral innate immunity. IRF-3 is also activated by linear ubiquitin chain assembly complex (LUBAC)-mediated linear ubiquitination, which triggers its interaction with Bcl-2 associated X protein (BAX) to cause mitochondrial activation and later in infection apoptotic death of virus-infected cells via the RIG-I-like receptor-induced IRF-3-mediated pathway of apoptosis (RIPA) pathway [8]. Recent studies have demonstrated that, besides its antiviral function, RIG-I plays a role in other cellular processes: RIG-I deficiency in mice induces progressive myeloproliferative disorder, enhances leukemic stemness, and promotes hepatocellular carcinogenesis [5, 9, 10].

In this study, we investigated whether RIG-I is involved in regulation of the stemness of hepatocellular carcinoma (HCC) cells and found that RIG-I deficiency resulted in upregulation of the expression of stem cell marker genes in human HCC cells. Our results showed that RIG-I-deficient HCC cells enhanced secretion of factors suppressing in vitro generation of DCs into their conditioned medium and induction of a phenotype of tumor-infiltrating DCs (TIDCs) with low DC markers in vivo. Those in vitro generated DCs and in vivo induced TIDCs under the influence of RIG-I-deficient HCC cells showed reduced mixed lymphocyte reaction (MLR), indicating RIG-I deficiency-induced immune tolerance which may lead to the development and progression of tumors. Our results also suggested that transforming growth factor (TGF)-β1 induction is involved in those RIG-I deficiency-induced tolerogenic changes.

## Methods

### Reagents

Recombinant human epidermal growth factor (EGF), basic fibroblast growth factor (bFGF), granulocyte-macrophage colony-stimulating factor (GM-CSF), and interleukin (IL)-4 were obtained from PeproTech Inc. (Rocky Hill, NJ, USA). Anti-human and anti-mouse monoclonal antibodies for cluster of differentiation (CD)11c, HLA-DR, major histocompatibility complex class II (MHCII), and CD86 labeled with fluorescein isothiocyanate (FITC) or R-phycoerythrin (PE) were purchased from eBioscience (San Diego, CA, USA). Anti-phospho (p)-Smad2 (S465), anti-Smad2, anti-p-Akt (T308), anti-Akt, and anti-RIG-I antibodies were obtained from Cell Signaling Technology (Beverly, MA, USA).

### Tumorsphere culture of human HCC cell lines

The human HCC cell lines SMMC-7721 and Bel-7402 were obtained from the Cell Bank of Type Culture Collection of the Chinese Academy of Sciences (CBTCCCAS, Shanghai, China) with catalog numbers TCHu 10 and TCHu 52, respectively, on March 2014. The cells were maintained as a monolayer in 10 cm-diameter dishes in Dulbecco’s modified Eagle’s medium (DMEM) supplemented with 10% fetal bovine serum (FBS), 100 IU/ml penicillin G, and 100 μg/ml streptomycin at 37 °C in 5% CO_2_ as described previously [11]. Authentification of both cell lines was performed by short tandem repeat (STR) DNA profiling analysis on March 2014 and December 2017. Mycoplasma contamination was not detectable in both cell lines by the two methods microbiological culture test and PCR test performed on March 2014 and December 2017. To form tumorspheres, SMMC-7721 and Bel-7402 cells (5 × 10^2^) were grown in 96-well ultra-low attachment culture plates in DMEM/Ham’s F-12 nutrient mixture (F-12) medium supplemented with 10 ng/ml of bFGF and 20 ng/ml of EGF for 10 days at 37 °C in 5% CO_2_ as described previously [11].

### Knockdown (ND) of RIG-I with short hairpin RNA (shRNA)

SMMC-7721 and Bel-7402 cell lines (2 × 10^7^ cells) that constitutively express a tetracycline-controlled transactivator were electroporated with pTRE2hyg–control shRNA plasmid or pTRE2hyg–RIG-I shRNA (RIG-Ish) plasmid using a Gene-Pulser II (Bio-Rad, Hercules, CA, USA) and selected with 0.5 mg/ml of hygromycin B, 0.5 μg/ml of puromycin, and 1 μg/ml of tetracycline (Merck–Calbiochem, La Jolla, CA, USA) for 3–4 weeks to obtain stably transfected cells as described previously [12]. The core sequences of the two RIG-Ishs, RIG-Ish1 and RIG-Ish2, and control shRNA inserted with flanked miR30a into the pTRE2hyg plasmid vector are shown in Additional file 1: Figure S1a.

### CM of RIG-I KD tumorsphere cultures of human HCC cell lines

Fresh tumorsphere cells (2 × 10^6^ cells/ml) of parental cell lines (NC), reference cell lines transfected with pTRE2hyg − control shRNA plasmid (NCsh) and RIG-1 KD cell lines transfected with pTRE2hyg − RIG-I shRNA plasmid (CRIG-Ish1 and CRIG-Ish2) were grown in 6-well ultra-low attachment culture plates in DMEM/F-12 medium supplemented with 100 IU/ml penicillin G, 100 μg/ml streptomycin, 10 ng/ml of bFGF and 20 ng/ml of EGF for 72 h and then the culture supernatant was collected as CM and stored at − 80 °C until use for experiments on generation of DCs and for determination of cytokines.

### ELISA of cytokines and prostaglandin E_2_ (PGE_2_)

Levels of IL-6, IL-12, IL-10, PGE_2_, vascular endothelial growth factor (VEGF), TGF-β1, and IFN-γ in the CM and culture supernatant were measured by ELISA assay kits (eBioscience) according to the manufacturer’s instructions. Briefly, to measure cytokines, each well of 96-well microtiter plates was coated with capture antibody by being incubated overnight at 4 °C. After blocking, the plates were incubated with standards or samples for 2 h at room temperature. The plates was then incubated sequentially with biotin-conjugated detection antibody, avidin-horseradish peroxidase and the substrate tetramethylbenzidine for 1 h, 30 min and 15 min, respectively, at room temperature. The enzyme reaction was stopped by addition of H_2_SO_4_, and the absorbance was read at 450 nm in an automatic plate reader. The samples were acidified with HCl before TGFß1 ELISA to activate latent TGFß1 to the immunoreactive form and then neutralized with NaOH. For PGE_2_ ELISA, each well of 96-well microtiter plates coated with antibody to anti-PGE_2_ monoclonal antibody were incubated with standards or samples in the presence of PGE_2_-alkaline phosphatase tracer and anti-PGE_2_ monoclonal antibody for 2 h at room temperature. After washing, the plates was incubated with the substrate p-nitrophenyl phosphate for 60 to 90 min at room temperature, and the absorbance was read at 420 nm in an automatic plate reader. PGE_2_ in the samples was purified with a SPE C18 cartridge prior to analysis according to the manufacturer’s instructions.

### Quantitative real-time PCR analysis

Total RNA was extracted from cells using RNAiso Plus (Takara Bio Inc., Kusatsu, Japan), and complementary DNA (cDNA) was synthesized with Moloney murine leukemia virus reverse transcriptase (Promega, Fitchburg, WI, USA) in accordance with the manufacturer’s protocol. The original amount of the specific transcripts was measured by real-time PCR with an SYBR Green PCR kit (Applied Biosystems, Thermo Fisher Scientific, Waltham, MA, USA) using the ABI Prism 7900 sequence detector (Applied Biosystems). The expression of specific genes was normalized against glyceraldehyde 3-phosphate dehydrogenase (GAPDH) gene expression. Primers used are listed in Additional file 2: Table S1.

### Western blot analysis

Western blotting was performed as described previously [13]. Briefly, cytoplasmic protein fractions of SMMC-7721 or Bel-7402 spheres cells were prepared using a NucBuster Protein Extraction kit (Novagen, Merck, Darmstadt, Germany). After protein quantification by the method of Bensadoun and Weinstein [14], cytoplasmic proteins were separated by 10% sodium dodecyl sulfate (SDS) − polyacrylamide gel electrophoresis and transferred to polyvinylidene difluoride (PVDF) membranes. After sequential incubation of the membranes with a primary antibody and a horseradish peroxidase-conjugated secondary antibody, the immunoblots were visualized using enhanced chemiluminescence (ECL) detection reagents (GE Healthcare Bio-Sciences, Piscataway, NJ, USA). Quantification of the detected bands was carried out, and the ratio of phosphorylated protein bands to total protein bands is shown in the figures. Since some studies have shown that phosphorylation of Akt at T308 is a more valuable biomarker for tumor progression than phosphorylation of Akt at S473, we used anti-p-Akt (T308) for Western blot analysis of Akt phosphorylation [15, 16].

### Flow cytometry

The surface molecule expression levels of DCs and TIDCs were analyzed using flow cytometry as described previously [17]. DCs and TIDCs were washed in a staining buffer (PBS/2%FBS) and incubated with FITC-labeled anti-HLA-DR antibody plus PE-labeled anti-CD11c antibody, FITC-labeled anti-MHCII antibody plus PE-labeled anti-CD11c antibody or FITC-labeled anti-CD86 antibody plus PE-labeled anti-CD11c antibody for 30 min at 4 °C. Negative control staining with appropriate isotype-matched control antibodies was included. Flow cytometric analysis was performed using a FACScan flow cytometer (BD Biosciences, San Jose, CA, USA).

### Generation of monocyte-derived DCs in vitro

Generation of monocyte-derived DCs was performed as described previously [18, 19]. Briefly, human peripheral blood mononuclear cells (PBMCs) were isolated from buffy coats of healthy donors provided by the Department of Transfusion Medicine, People’s Hospital of Rizhao, China using Ficoll gradient. CD14^+^ monocytes were purified from PBMCs with anti-CD14 antibody-magnetic microbeads (Miltenyi Biotec, Bergisch Gladbach, Germany). To generate dendritic cells, CD14^+^ monocytes (2 × 10^6^ cells/ml) were cultured in RPMI-1640 medium supplemented with 10% FBS, 100 IU/ml penicillin G, 100 μg/ml streptomycin, 40 ng/ml GM-CSF and 40 ng/ml IL-4 for 14 days at 37 °C in 5% CO_2_. HLA-DR and CD86 expression on DCs was then assessed by flow cytometry after gating on a CD11c^+^ population. This study employing CD14^+^ monocytes from PBMCs of healthy donors was approved by the Medical Ethics Committee of Jining Medical College and the Medical Ethics Committee of People’s Hospital of Rizhao, and written informed consent was obtained from all donors.

### MLR

DC generation culture (4 × 10^4^ cells) treated with 20 μg/ml of mitomycin C for 2 h and allogeneic CD3^+^ T cells (4 × 10^5^) purified from PBMCs using human T cell enrichment columns (R&D Systems, Minneapolis, MN, USA) were co-cultured in U-bottom 96-well plates in RPMI-1640 medium supplemented with 10% FBS, 100 IU/ml penicillin G and 100 μg/ml streptomycin for 72 h at 37 °C in 5% CO_2_. The CD3^+^ T cells were then pulse-labeled with [^3^H] thymidine (1.0 μCi/well, 5.0 Ci/mmol) for 16 h and harvested on glass fiber filters using an automated cell harvester as described previously [17]. The amount of [^3^H] thymidine incorporated was measured using a liquid scintillation counter. This study employing CD3^+^ T cells from PBMCs of healthy donors was approved by the Medical Ethics Committee of Jining Medical College and the Medical Ethics Committee of People’s Hospital of Rizhao, and written informed consent was obtained from all donors.

### Animal studies

Forty-eight male BALB/c nude mice (6 weeks of age), twelve BALB/c mice (6 weeks of age) and twelve male C57 BL/6 mice (6 weeks of age) were purchased from the Shandong University Laboratory Animal Centre (Jinan, China). All animal experiments were conducted in accordance with the recommendations in the Guide for the Care and Use of Laboratory Animals from the National Institute of Health and approved by the Animal Care and Use Committee at Jining Medical College. All mice were guaranteed to be free of particular pathogens by the Laboratory Animal Centre at Jining Medical College, housed under constant temperature of 22 ± 2 °C with a 12-h light/dark cycle and fed standard mouse chow and water ad libitum throughout the study. BALB/c nude mice were randomly divided into four groups of two mice each: SMMC-7721 NCsh tumorsphere cell-injected, SMMC-7721 CRIG-Ish tumorsphere cell-injected, Bel-7402 NCsh tumorsphere cell-injected, and Bel-7402 CRIG-Ish tumorsphere cell-injected groups. Fresh CRIG-Ish and NCsh tumorspheres of both cell lines were collected in sterile DMEM/F-12 medium without FBS and then 2 × 10^6^ cells were injected into the armpits of each mice. The injected mice were housed two per cage. The lengths of major and minor axes of the tumors were measured every week thereafter for 4 weeks. The tumor volume was calculated by using the following formula: (length of minor axes)^2^ × (length of major axes)/2. The experiment was independently repeated six times. At 28 days after tumor inoculation, mice were sacrificed with the method of euthanasia using CO_2_ inhalation, and the tumor tissues from two mice were collected and combined. TIDCs were then isolated and purified using CD11c ultrapure microbeads (Miltenyi Biotec) as described previously [20] to investigate the effect of RIG-I-deficient HCC cells on phenotypic change of TIDCs. Similarly, splenic DCs of two normal BALB/c mice were prepared. Expression levels of MHCII and CD86 on those splenic DCs and TIDCs and the level of TGF-β1 in lysates of tumors were measured as described in the sections of “Flow cytometry” and “ELISA of cytokines and PGE_2_”, respectively. Preparation of tumor lysates and their protein assay were performed by using T-PER tissue extraction reagent (Pierce Biotechnology, Rockford, IL, USA) and BCA protein assay reagent (Pierce), respectively, according to the manufacturer’s instructions. TIDCs (1 × 10^4^ cells) irradiated (30 Gy) as described previously [21] were co-cultured for 72 h with allogenic CD3^+^ T cells (1 × 10^5^ cells) purified from the spleens of C57 BL/6 mice using a mouse T Lymphocyte Enrichment Set (BD Biosciences) as described in the section of “MLR”. T cell proliferation and the levels of TGF-β1 and IFN-γ in the co-culture supernatants were measured as described in the sections of “MLR” and “ELISA of cytokines and PGE_2_”, respectively.

### Akt siRNA transfection

CRIG-Ish1 and NCsh SMMC-7721 cells (5 × 10^5^) were plated in 35-mm-diameter dishes and cultured in DMEM supplemented with 10% FBS, 100 IU/ml penicillin G and 100 μg/ml streptomycin at 37 °C in 5% CO_2_. After 24 h, the cells were washed once with a fresh medium and transfected for 72 h with 50 nM Akt1/2 small interfering RNA (siRNA) (sc-43,609, Santa Cruz Biotechnology, Dallas, TX, USA) according to manufacturer’s instructions. Akt protein level in Akt siRNA-treated CRIG-Ish1 SMMC-7721 cells was greatly reduced (Additional file 1: Figure S1b).

### Data analysis

The results are expressed as means and standard deviation (SD) of more than three independent experiments. Student’s *t*-test was used to analyze data in two groups. Tukey’s test was used for multiple comparison analysis testing in one-way analysis of variance (ANOVA). A *P* value less than 5% was regarded as statistically significant.

## Results

### Upregulation of the expression of stem cell marker genes in RIG-I-KD HCC spheres

**Fig. 1:**
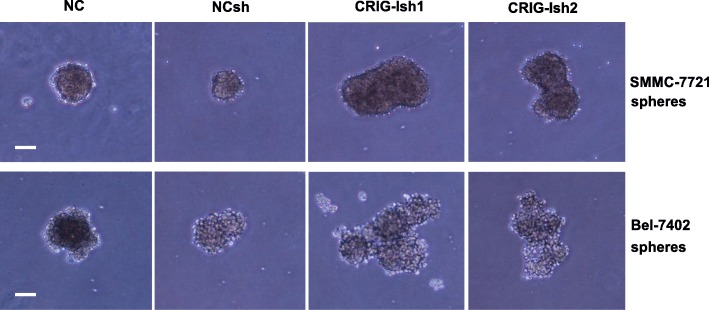
Tumorsphere formation is enhanced by RIG-I KD. RIG-I knocked-down cells (CRIG-Ish1 and CRIG-Ish2) and controls (NC and NCsh) of SMMC-7721 and Bel-7402 cell lines were grown in 96-well ultra-low attachment culture plates for 10 days. The tumorspheres formed were observed under a microscope. Scale bars, 100 μm

**Fig. 2:**
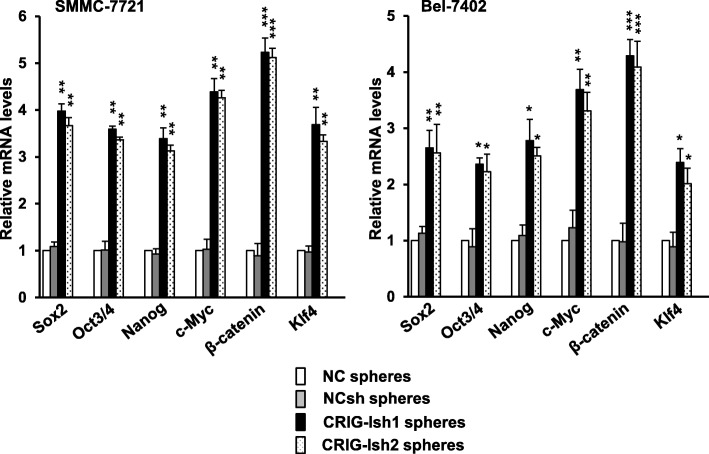
The mRNA levels of stem cell markers in tumorspheres are increased by RIG-I KD. RIG-I knocked-down and control SMMC-7721 and Bel-7402 cell lines were grown in 6-well ultra-low attachment culture plates to form spheres for 10 days. Expression of stem cell marker genes was determined by real-time PCR. The level of each gene mRNA was normalized against GAPDH mRNA level and expressed as a ratio to the value of NC spheres. The values are presented as means ± SD (*n* = 3). *P < 0.05, **P < 0.01 and ***P < 0.001 vs. NCsh spheres

Since three-dimesional sphere cell aggregates of human HCC cell lines have been reported to possess properties of liver cancer stem-like cells [11], we used sphere cultures of the human HCC cell lines SMMC-7721 and Bel-7402 in this study. To investigate the role of RIG-I in regulation of the stemness of HCC cell lines, we established RIG-I-deficient human SMMC-7721 and Bel-7402 cell lines that were stably transfected with RIG-I shRNA plasmid (Additional file 1: Figure S1a). RIG-I protein levels in the RIG-I KD human SMMC-7721 and Bel-7402 cell lines were greatly reduced (Additional file 1: Figure S1c). Tumorsphere formation after 10 days of culture was compared among NC, NCsh, CRIG-Ish1, and CRIG-Ish2. CRIG-Ish1 and CRIG-Ish2 of the SMMC-7721 cell line formed larger spheres than did NC and NCsh of the same cell line (Fig. 1, upper panel). Similarly, spheres of Bel-7402 CRIG-Ish1 and CRIG-Ish2 grew more rapidly than did spheres of NC and NCsh of the same cell line (Fig. 1, lower panel). To assess the stemness of the RIG-I-deficient HCC cell line spheres, expression of genes considered as stem cell markers (Sox2, Oct3/4, Nanog, c-Myc, β-catenin, and Klf4) was determined. The expression of all of the stemness-related genes was significantly upregulated in RIG-I-deficient spheres of SMMC-7721 and Bel-7402 cell lines compared with the expression of those genes in NC and NCsh spheres of the same cell line (Fig. 2). Expression of β-catenin gene was most markedly upregulated in RIG-I-deficient tumorspheres of both cell lines (Fig. 2).

### Inhibition of in vitro generation of DCs and of MLR by the CM of RIG-I-deficient HCC spheres

**Fig. 3:**
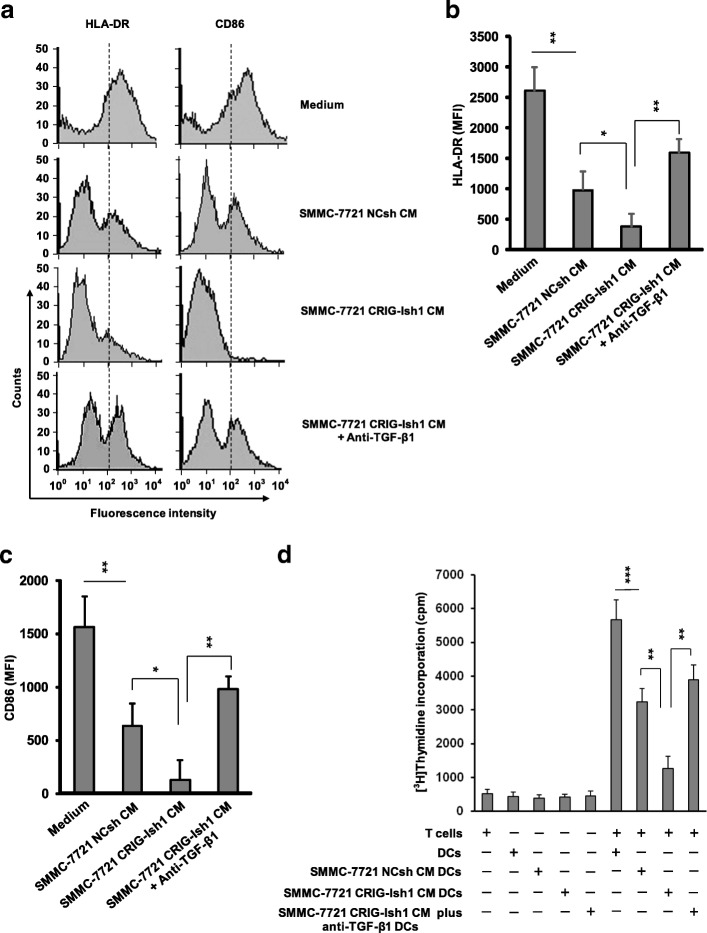
Inhibition of DC generation by the CM of RIG-I-dificient tumorspheres and its restoration by anti-TGF-β1. **a** − **c** CD14^+^ monocytes were cultured for 14 days in a medium containing 40 ng/ml of GM-CSF and 40 ng/ml of IL-4 in the presence and absence of 1 μg/ml of TGF-β1 neutralizing antibody. Half of the medium was replaced every day with the CM of RIG-I KD or reference SMMC-7721 tumorspheres or a fresh medium, to each of which 40 ng/ml of GM-CSF, 40 ng/ml of IL-4 and 1 μg/ml of TGF-β1 were added if necessary, starting 1 day after seeding. HLA-DR and CD86 expression on generated DCs was assessed by flow cytometry after gating on a CD11c^+^ population. DCs with fluorescence intensity higher than the level of the dotted line in (**a)** are regarded as positive populations. **d** Mitomycin C-treated DCs were co-cultured with allogeneic CD3^+^ T cells for 72 h, and the proliferation of T cells was then determined by measuring [^3^H] thymidine uptake during the next 16 h of culture. The values are presented as means ± SD (*n* = 3). *P < 0.05, **P < 0.01 and ***P < 0.001

The CM of RIG-I-deficient stemness-high SMMC-7721 HCC spheres was prepared after reculturing the sphere cells for 3 days at a high density (2 × 10^6^ cells/ml) and was used to investigate whether the microenvironment of CSCs affects in vitro differentiation and function of DCs. Human peripheral blood monocytes were incubated for 14 days with GM-CSF and IL-4 to generate DCs in the presence and absence of the CM of CRIG-Ish or NCsh tumorspheres. Expression levels of the DC markers HLA-DR and CD86 were reduced by addition of the CM of NCsh spheres and more greatly reduced by addition of the CM of CRIG-Ish spheres (Fig. 3a, b and c). The function of those DCs was determined by measuring MLR. The cultures containing DCs generated in the presence and absence of the CM of NCsh spheres or the CM of CRIG-Ish spheres were treated with mitomycin C and co-cultured with allogeneic CD3^+^ T cells. Among T cells co-cultured with CRIG-Ish spheres-CM-treated DCs, NCsh spheres-CM-treated DCs and CM-untreated DCs, T cells co-cultured with CM-untreated DCs exhibited the highest rate of proliferation, and T cells co-cultured with CRIG-Ish spheres-CM-treated DCs showed the lowest rate of proliferation, indicating RIG-I deficiency-induced immune tolerance (Fig. 3d).

### Upregulation of TGF-β1 secretion in RIG-I-deficient HCC spheres

**Fig. 4:**
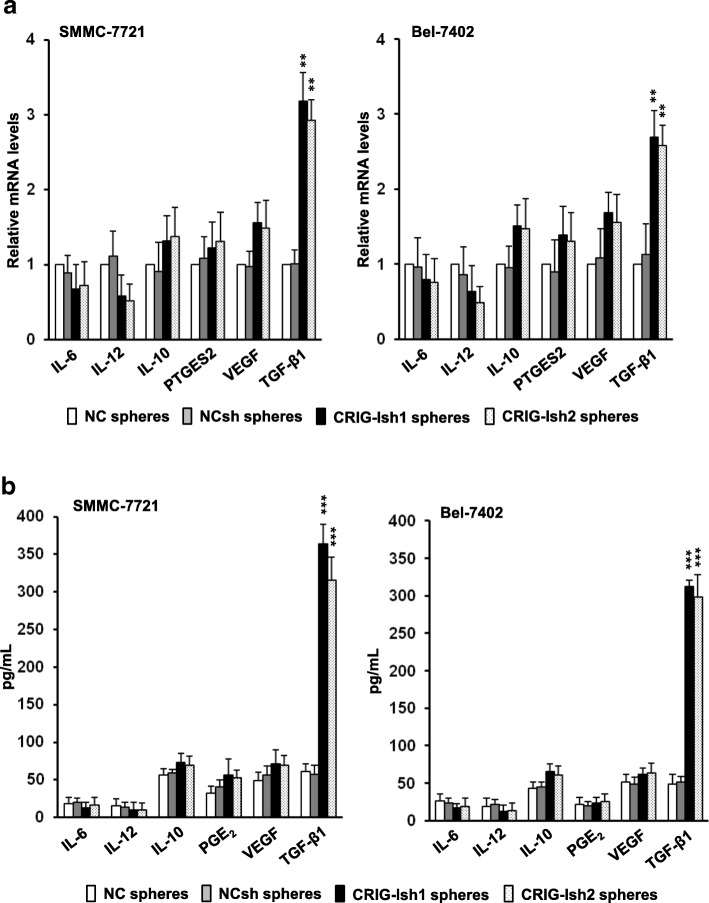
TGF-β1 expression in tumorspheres is enhanced by RIG-I KD. **a** Tumorspheres of RIG-I knocked-down and control SMMC-7721 and Bel-7402 cell lines that had formed were collected, and the cells (2 × 10^6^ cells/ml) were again grown in 6-well ultra-low attachment culture plates in DMEM/F-12 medium supplemented with 10 ng/ml of bFGF and 20 ng/ml of EGF for 72 h, and then the mRNA levels of cytokines and PTGES2 in the tumorsphes were determined by real-time PCR. **b** The levels of cytokine proteins and PGE_2_ in the culture supernatants were analyzed by ELISA. The values are presented as means ± SD (n = 3). **P < 0.01 and ***P < 0.001 vs. NCsh spheres

Human HCC cell lines are known to secrete various kinds of immunosuppressive cytokines and other molecules [22]. We determined messenger RNA (mRNA) levels in the tumorspheres and protein levels secreted in the conditioned medium of several typical factors. Gene expression and protein secretion levels of TGF-β1 in CRIG-Ish spheres of SMMC-7721 cells were markedly higher than gene expression and protein secretion levels of TGF-β1 in NC and NCsh spheres of the same cell line, respectively, but mRNA and protein levels of other factors including IL-12, IL-10, prostaglandin E synthase 2 (PTGES2)/PGE_2_, and VEGF did not differ among CRIG-Ish, NC and NCsh spheres (Fig. 4a and b, left panels). Similarly, only TGF-β1 gene expression and protein secretion levels in CRIG-Ish spheres of Bel-7402 cell line were significantly higher than gene expression and protein secretion levels of the cytokine in NC and NCsh spheres of the same cell line, respectively (Fig. 4a and b, right panels).

### A critical role of TGF-β1 in the CM of RIG-I-deficient HCC spheres in generation of DCs

To test whether increased TGF-β1 in the CM of CRIG-Ish spheres plays a role in inhibition of the expression of DC markers and MLR, an anti-TGF-β1 neutralizing antibody was added to the CRIG-Ish-spheres CM-treated culture for generation of DCs. The expression of DC markers and MLR suppressed by CRIG-Ish-spheres CM of the SMMC-7721 cell line were greatly restored by simultaneous addition of an anti-TGF-β1 neutralizing antibody to the DC generation cultures (Fig. 3a, b, c and d).

### Impaired expression of DC surface markers on TIDCs and defective MLR of TIDCs from RIG-I-deficient HCC tumors in vivo

**Fig. 5:**
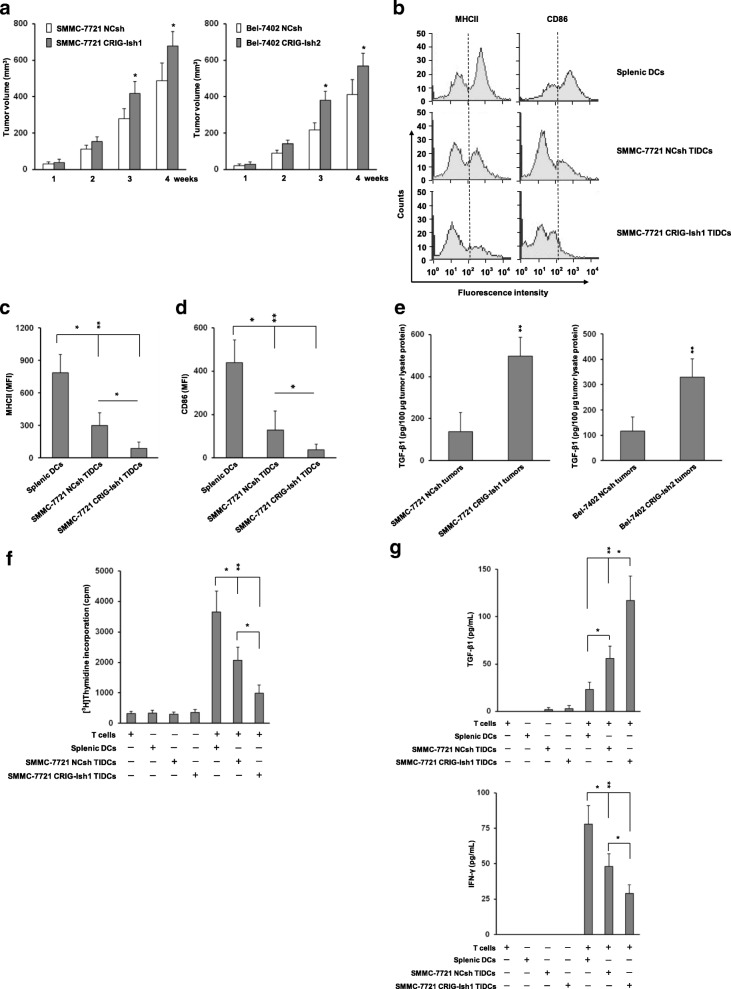
Induction of tolerogenic TIDCs in RIG-I-deficient HCC tumors in vivo. **a** RIG-I-deficient or reference tumorsphere cells of SMMC-7721 or Bel-7402 cell lines (2 × 10^6^) were injected into armpits of BALB/c nude mice. The tumor volume was calculated weekly from the lengths of major and minor axes of the tumors. **b** − **d** At 28 days after tumor inoculation, TIDCs and splenic DCs of normal BALB/c mice were isolated and purified using CD11c microbeads. MHCII and CD86 expression on those TIDCs and splenic DCs was assessed by flow cytometry. TIDCs with fluorescence intensity higher than the level of the dotted line in (**b)** are regarded as positive populations. **e** The level of TGF-β1 in tumor lysates was determined by ELISA. **f** Irradiated TIDCs or irradiated normal mice splenic DCs were co-cultured with allogenic CD3^+^ T cells from C57 BL/6 mice for 72 h, and the proliferation of T cells was determined by measuring [^3^H] thymidine uptake during the next 16 h of culture. **g** The levels of TGF-β1 and IFN-γ in co-culture supernatants were analyzed by ELISA. The values are presented as means ± SD (*n* = 12 for **a** and *n* = 6 for **b**-**g**). *P < 0.05 and **P < 0.01

A recent study by Scarlett et al. showed that DCs may alter their role from being immunostimulatory to immunosuppressive at advanced stages of tumor progression by being replaced with an immunosuppressive DC phenotype with lower levels of MHCII [3]. The impact of RIG-I-deficient HCC cells with high stemness on MHCII and CD86 expression of TIDCs in vivo was investigated. CRIG-1sh or NCsh HCC sphere cells (2 × 10^6^) of SMMC-7721 and Bel-7402 cell lines were injected into armpits of nude mice. No adverse events were observed and all treated mice survived throughout the experiment. Figure 5a shows the growth of those tumors measured weekly after inoculation. SMMC-7721 and Bel-7402 CRIG-Ish tumors showed higher growth rates than those of SMMC-7721 and Bel-7402 NCsh tumors, respectively (*n* = 12). The body weights of SMMC-7721 NCsh and CRIG-1sh sphere cells-injected mice were 16.09 ± 1.04 g and 16.38 ± 0.95 g before injection, respectively and 18.80 ± 1.12 g and 18.73 ± 0.98 g at day 28 after injection, respectively. No significant difference was observed between the two groups at either point in time. The body weights of Bel-7402 NCsh and CRIG-1sh sphere cells-injected mice were 16.50 ± 0.97 g and 16.33 ± 1.04 g before injection, respectively and 19.63 ± 1.18 g and 19.02 ± 1.33 g at day 28 after injection, respectively. Similarly, no significant difference was observed between them at either point in time. SMMC-7721 tumor tissues from two mice were then collected and combined 28 days after inoculation, and TIDCs were prepared. Expression of the DC markers MHCII and CD86 on NCsh and CRIG-Ish tumor TIDCs was downregulated compared with the expression of both markers on splenic DCs, CRIG-Ish tumor TIDCs exhibiting significantly lower expression levels of MHCII and CD86 than those of NCsh tumor TIDCs (Fig. 5b, c and d, *n* = 6). The SMMC-7721 and Bel-7402 CRIG-Ish tumors had much higher TGF-β1 contents than those of the SMMC-7721 and Bel-7402 NCsh tumors, respectively (Fig. 5e, *n* = 6). Irradiated SMMC-7721 CRIG-Ish tumor TIDCs less strongly stimulated proliferation of allogeneic T cells than did irradiated SMMC-7721 NCsh tumor TIDCs, indicating RIG-I deficiency-induced immune tolerance, although both TIDCs induced lower MLR than did irradiated splenic DCs (Fig. 5f, *n* = 6). The concentration of TGF-β1 secreted during culture of allogeneic T cell proliferation was highest in the supernatants of cultures containing SMMC-7721 CRIG-Ish tumor TIDCs and lowest in the supernatants of cultures containing splenic DCs (Fig. 5g, upper panel, *n* = 6). The amount of IFN-γ produced was in the reverse order: highest in the supernatants of cultures containing splenic DCs and lowest in the supernatants of cultures containing SMMC-7721 CRIG-Ish tumor TIDCs (Fig. 5g, lower panel, *n* = 6).

### Upregulation of TGF-β1-induced phosphorylation of Smad2 and Akt and association of Smad2/p-Smad2 and Akt/p-Akt in RIG-I-deficient HCC spheres

**Fig. 6:**
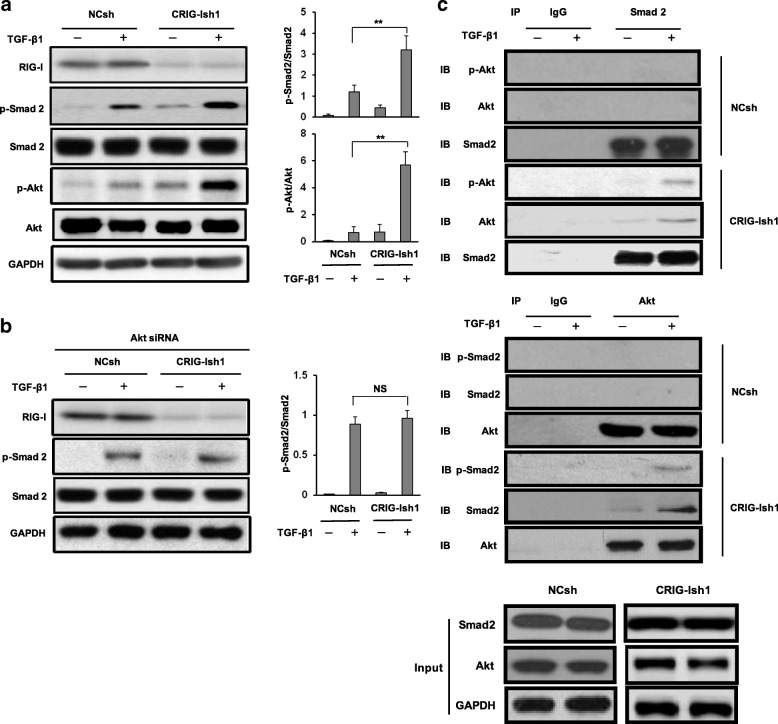
TGF-β/Akt/Smad signaling pathway in HCC spheres is activated by RIG-I KD. **a** RIG-I knocked-down and reference SMMC-7721 tumorspheres were stimulated with 2 ng/ml of TGF-β1 for 1 h. Western blot analysis was performed using the indicated antibodies. **b** RIG-I knocked-down and reference SMMC-7721 cells were transfected with Akt1/2 siRNA. Akt-deficient tumorspheres were stimulated with TGF-β1 in the same manner as (**a**), and Western blot analysis was performed using the indicated antibodies. **c** RIG-I knocked-down and reference SMMC-7721 tumorspheres were stimulated with TGF-β1 in the same manner as (**a**). Smad2/p-Smad2 and Akt/p-Akt interaction was detected by immunoprecipitation with anti-Smad2 antibody (upper panel) or anti-Akt antibody (lower panel), IgG being used as a negative control antibody. Levels of GAPDH protein were monitored as a loading control. The values are presented as means ± SD (n = 3). **P < 0.01

It has been reported that TGF-β1 auto-upregulates its own mRNA expression, resulting in an increase in its own secretion [23]. It is conceivable that signal transduction of TGF-β1 in HCC tumor spheres is also influenced by RIG-I KD, thereby contributing to increased secretion of TGF-β1. We therefore investigated whether signal transduction of TGF-β1 in HCC tumor spheres is influenced by RIG-I KD. Since Smad2 and Akt are key components in Smad and non-Smad pathways of TGF-β signaling, respectively [24], activation of the both proteins in RIG-I-deficient and reference SMMC-7721 spheres stimulated with TGF-β was determined. Although both Smad2 and Smad3 are prominent components of the Smad pathway and have overlapping and distinct roles in TGF-β signaling for transcriptional activation depending on the target gene and cellular context, we employed Smad2 rather than Smad3 because the latter has also been involved in transcriptional repression of some genes more frequently than the former [25]. TGF-β-induced phosphorylation levels of Smad2 and Akt in CRIG-Ish spheres were higher than TGF-β-induced phosphorylation levels of Smad2 and Akt in NCsh spheres, respectively, the increase in Akt phosphorylation being more marked than the increase in Smad2 phosphorylation (Fig. 6a). To explore an interaction of Akt/p-Akt with Smad2/p-Smad2, *AKT* gene expression in CRIG-Ish and NCsh spheres was knocked down with siRNA. Akt protein level in Akt siRNA-treated cells was reduced to 13.3 ± 4.0% of untreated cells (Additional file 1: Figure S1b). The TGF-β-induced Smad2 phosphorylation level in CRIG-Ish spheres was reduced to the level in NCsh spheres by Akt KD (Fig. 6b). We then performed immunoprecipitation experiments of tumorsphere cytoplasmic proteins using the anti-Smad2 antibody to detect an association between Smad2 and Akt. Akt and p-Akt were co-immunoprecipitated along with Smad2 in cytoplasmic proteins of CRIG-Ish spheres but not in cytoplasmic proteins of NCsh spheres, the amount of both co-immunoprecipitated Akt and p-Akt being increased by TGF-β stimulation without a change in the amount of immunoprecipitated Smad2 (Fig. 6c, upper panel). Similarly, Smad2 and p-Smad2 were co-immunoprecipitated along with Akt in cytoplasmic proteins of CRIG-Ish spheres but not in cytoplasmic proteins of NCsh spheres by immunoprecipitation of tumorsphere cytoplasmic proteins with the anti-Akt antibody (Fig. 6c, lower panel). The amount of both co-immunoprecipitated Smad2 and p-Smad2 was increased by TGF-β stimulation without a change in the amount of immunoprecipitated Akt (Fig. 6c, lower panel).

## Discussion

Although a switch from immune stimulatory DCs to immune suppressive DCs occurs during tumor progression, little is known about the influence of CSCs on the shift of DCs as well as on the generation of DCs. The present study demonstrated that RIG-I-deficient stemness-high human HCC cells secreted factors including a markedly increased amount of TGF-β1, suppressed in vitro generation of DCs and induced a tolerogenic TIDC phenotype with lower levels of MHCII in vivo. Those results suggest that CSCs play a role in triggering the phenotypic change of DCs in tumor progression. Since the suppression of in vitro DC generation caused by the CM of RIG-I-deficient human HCC cells was ameliorated with a neutralizing anti-TGF-β1 antibody and since tumors derived from RIG-I-deficient human HCC cells contained a large amount of TGF-β1, our results also suggest that TGF-β1 is involved in suppression of DC generation and in the phenotypic change of TIDCs. Those are unexpected findings because there are few reports in which increased production of TGF-β in liver CSCs is described and because there have been only a few studies on the effect of TGF-β on induction of stimulatory or suppressive DCs [26, 27] even though serum levels of TGF-β are increased in HCC patients [28] and TGF-β exerts systemic immune suppression and inhibits host immune surveillance [29, 30]. In this study, the in vitro differentiation culture of human DCs was exposed to the CM of RIG-I-deficient stemness-high human HCC cells to mimic a CSC tumor microenvironment. Although the method requires two steps, the results with the in vitro human DC cultures consistent with induction of a tolerogenic TIDC phenotype in RIG-I-deficient human HCC tumors in nude mice imply not only the replacement or reduction of animal experiments but also clinical relevance.

The mechanism underlying the upregulation of TGF-β1 expression in RIG-I-deficient HCC cells remains to be clarified. It is possible, however, that interaction of RIG-I and STAT3 is involved. A recent study has shown that foreign RNA-unliganded RIG-I physically associates with STAT3, thereby impeding the association of STAT3 with JAK1 while facilitating the association of STAT3 with SHP2 to reduce the level of p-STAT3 [31]. STAT3 is activated by growth factors such as EGF and bFGF [32, 33], which were included in the medium for culture of the HCC cell line spheres in our experiments, and therefore p-STAT3 may accumulate in the RIG-I-deficient HCC cells. Since STAT3 is known to be involved in TGF-β1 gene expression [34], elevated levels of p-STAT3 could upregulate TGF-β1 gene expression under the condition of RIG-I deficiency. Moreover, TGF-β induces phosphorylation of STAT3 in both Smad-independent and -dependent manners, presumably causing further upregulation of TGF-β1 gene expression [35].

Since TGF-β1 auto-upregulates its own mRNA expression and secretion [23] and since phosphorylation of receptor-activated Smads including Smad2 is a key step in Smad-mediated transcription [36], enhancement of TGF-β1-induced Smad2 phosphorylation in RIG-I-deficient tumorspheres might also contribute to the increased mRNA expression and secretion of TGF-β1. TGF-β1-induced phosphorylation of Akt, a key component in the non-Smad pathway of TGF-β signaling, was also augmented in RIG-I-deficient tumorspheres. KD of Akt gene expression abolished TGF-β1-induced phosphorylation of Smad2 that was enhanced in RIG-I-deficient tumorspheres to the level of TGF-β1-induced Smad2 phosphorylation in the reference tumorspheres (Fig. 6b), indicating Akt-dependent augmentation of Smad2 phosphorylation. The association between Smad2/p-Smad2 and Akt/p-Akt observed in RIG-I-deficient tumorspheres but not in the reference tumorspheres (Fig. 6c) could be involved in the enhancement of Smad2 phosphorylation, although little has been reported on the association between Smad2/p-Smad2 and Akt/p-Akt. One possible mechanism for the Akt dependency of enhanced TGF-β1-induced Smad2 phosphorylation is that the association of Akt/p-Akt with Smad2/p-Smad2 may protect Smad2/p-Smad2 from phosphatase-mediated dephosphorylation and/or ubiquitination-dependent degradation. One such protein is chloride intracellular channel 4, which associates with p-Smad2 and p-Smad3, protecting them from dephosphorylation by phosphatases [37]. PPM1A/PP2Cα has been identified as a Smad2/3 phosphatase and abrogates the signaling activity of Smad2/3 [38]. The E3 ubiquitin ligase Arkadia has been shown to ubiquitinate p-Smad2/3, leading to their proteosomal degradation [39].

RIG-I deficiency-enhanced tumor stemness has been reported in leukemia and was detected in this study in HCC [5]. Although RIG-I is a pattern recognition receptor that detects cytoplasmic viral nucleic acids followed by not only inducing IFN production but also triggering apoptotic death in virus-infected cells [8], other intrinsic biological activities of viral RNA-unliganded RIG-I have been reported. RIG-I deficiency promotes HCC carcinogenesis, suggesting that RIG-I is a tumor suppressor in HCC [10]. RIG-I interacts with STAT3 to inhibit STAT3 activation in CD4^+^ T cells, which contributes to the maintenance of a regulatory T cell (Treg)/T helper cell 17 (Th17) balance [31]. RIG-I has been shown to play a critical role in negative regulation of myelopoiesis since RIG-I-targeted mice developed a progressive myeloproliferative disorder partly through reduced expression of IFN consensus sequence-binding protein, a major transcription factor regulating myeloid cell differentiation [9]. A recent study has shown that foreign RNA-unliganded RIG-I inhibits the Src-facilitated activation of Akt-mammalian target of rapamycin (mTOR) in acute myeloid leukemia cells by competing with Src for recognition of Akt [5]. The RIG-I-induced inhibition of Akt activation was shown to contribute to RIG-I’s negative regulation of oncogenic cytokine-stimulated proliferation of primary myeloid progenitors and also to its negative regulation of in vivo maintenance of leukemia stemness [5]. A critical role of the PI3K/Akt signaling pathway in the induction and maintenance of other CSCs or cancer stem-like cells has also been reported [40–42]. Akt is activated by TGF-β to be a key component in the non-Smad pathway of TGF-β signaling in TGF-β-responsive cells [24, 43]. The results in the present study showed that TGF-β1-induced Akt phosphorylation was markedly enhanced in the RIG-I-deficient HCC cell lines. Taken together, the results suggest that the large amount of TGF-β1 secreted from the RIG-I-deficient HCC cell lines augments stemness of the cells via Akt activation.

RIG-I-deficient HCC cells showed a higher growth rate than that of the reference HCC cells both in vitro and in vivo (Figs. 1 and 5a) even though RIG-I-deficient HCC cells secreted a larger amount of TGF-β1. Although TGF-β is known to exert tumor-promoting effects in the onset and progression of liver cancer, TGF-β1 shows an inhibitory effect or no effect on the proliferation of HCC cells depending on the cell line. A study by Dzieran et al. using monolayer cultures of ten HCC cell lines showed that the cell lines can be divided into at least two groups with respect to TGF-β sensitivity: groups responsive and unresponsive to TGF-β-induced apoptosis and proliferation inhibition [44]. The former group, including PLC, HepG2 and HuH7, shows low levels of TGF-β mRNA expression and strong transcriptional Smad3 activity and might represent an early stage of liver carcinogenesis. In contrast, the latter group, including HLE, HLF and FLC-4, shows high levels of TGF-β mRNA expression and reduced Smad3 signaling and might represent a late stage of liver carcinogenesis. Huang et al. reported that exogenous addition of TGF-β1 inhibited the proliferation of SMMC-7721 HCC cells in a monolayer culture [45]. Therefore, TGF-β1 may not be involved in the higher growth rate of RIG-I-deficient HCC cells, but increased stemness, factors other than TGF-β1 or immunosuppression revealed in this study might contribute to it alone or in a collaborative manner.

## Conclusions

**Fig. 7:**
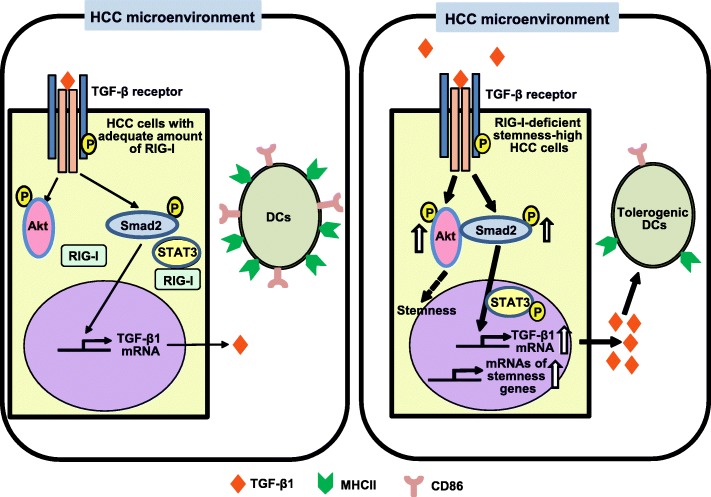
Graphical abstract of the main findings of this study and of related existing data. Main findings of this study: upregulated expression of stemness-related genes and TGF-β1 genes in RIG-I-deficient HCC cells, suppression of the generation of DCs and induction of an immunosuppressive DC phenotype with low levels of MHCII possibly by TGF-β1 secreted into the RIG-I-deficient HCC microenvironment, enhancement by RIG-I deficiency of TGF-β1-induced phosphorylation of the key TGF-β1 signaling components Smad2 and Akt in HCC cells, suggesting its involvement in autoinduction-mediated amplification of TGF-β1 mRNA expression and secretion, Akt dependence of the augmented TGF-β1-induced phosphorylation of Smad2, and induction by RIG-I deficiency of Smad2/p-Smad2 and Akt/p-Akt association in HCC cells. Our study suggests that CSCs are involved in DC-mediated immune tolerance. Related existing data: physical association of RIG-I with STAT3 to reduce the level of p-STAT3 [31], TGF-β-induced phosphorylation of STAT3 [35], involvement of STAT3 in TGF-β1 gene expression [34], and possible critical role of Akt in the induction and maintenance of CSCs or cancer stem-like cells [41–43]

Our results demonstrate that RIG-I deficiency in HCC cells induced their stemness, TGF-β1 secretion and tolerogenic TIDCs in the tumor microenvironment and reduced generation of monocyte-derived DCs and the results suggest involvement of CSCs in DC-mediated immune tolerance which enables the development and progression of tumors, increased TGF-β1 being possibly involved at least partly in those events. RIG-I deficiency also enhanced TGF-β1-induced phosphorylation of the key TGF-β1 signaling components Smad2 and Akt in HCC cells and induced the association of Smad2/p-Smad2 and Akt/p-Akt that was enhanced by TGF-β1. The augmentation of TGF-β1-induced Smad2 phosphorylation in RIG-I deficient HCC cells was dependent on the presence of Akt and may be involved in autoinduction-mediated amplification of TGF-β1 mRNA expression and secretion. The results are illustrated in the graphic abstract of Fig. 7.

## Additional files

Additional file 1: **Figure S1.** RIG-I knocked-down SMMC-7721 and Bel-7402 cell lines and Akt knocked-down SMMC-7721 cell line. (PPTX 64 kb)

Additional file 2: **Table S1.** The primer sequences for quantitative real-time PCR used in the study. (DOCX 14 kb)

Additional file 3: **Checklist S1.** NC3Rs ARRIVE Guideline Checklist (MZ et al.). (DOCX 658 kb)

## Abbreviations

Akt: Protein kinase B
ANOVA: Analysis of variance
bFGF: Basic fibroblast growth factor
CD: Cluster of differentiation
CM: Conditioned medium
CRIG-Ish: RIG-1-deficient cell line transfected with pTRE2hyg − RIG-I shRNA plasmid
CSCs: Cancer stem cells
DCs: Dendritic cells
DMEM: Dulbecco’s modified Eagle’s medium
DMEM/F-12: DMEM/Ham’s F-12 nutrient mixture
EGF: Epidermal growth factor
FBS: Fetal bovine serum
FITC: Fluorescein isothiocyanate
GAPDH: Glyceraldehyde 3-phosphate dehydrogenase
GM-CSF: Granulocyte-macrophage colony-stimulating factor
HCC: Hepatocellular carcinoma
HLA: Human leukocyte antigen
IFN: Interferon
IL: Interleukin
IRF: Interferon regulatory factor
JAK: Janus kinase
KD: Knockdown
MHC: Major histocompatibility complex
mRNA: Messenger RNA
NC: Parental cell line
NCsh: Reference cell line transfected with pTRE2hyg − control shRNA plasmid
p: Phospho
PBMCs: Peripheral blood mononuclear cells
PCR: Polymerase chain reaction
PE: R-phycoerythrin
PGE_2_: Prostaglandin E_2_
PTGES2: Prostaglandin E synthase 2
RIG-I: Retinoic acid-inducible gene I
RIG-Ish: RIG-I shRNA
SD: Standard deviation
shRNA: Short hairpin RNA
siRNA: Small interfering RNA
STAT: Signal transducers and activator of transcription
TGF: Transforming growth factor
TIDCs: Tumor-infiltrating dendritic cells
VEGF: Vascular endothelial growth factor
