# Supplementary material for: Induction of tolerogenic dendritic cells by activated TGF-β/Akt/Smad2 signaling in RIG-I-deficient stemness-high human liver cancer cells
Source: BMC Cancer. 2019 May 14;19:439. doi: 10.1186/s12885-019-5670-9 (PMC6515680; doi:10.1186/s12885-019-5670-9)
Supplement: Supplementary file 1 — Figure S1. RIG-I knocked-down SMMC-7721 and Bel-7402 cell lines and Akt knocked-down SMMC-7721 cell line. (PPTX 64 kb) [file 12885_2019_5670_MOESM1_ESM.pptx]

## Slide 1
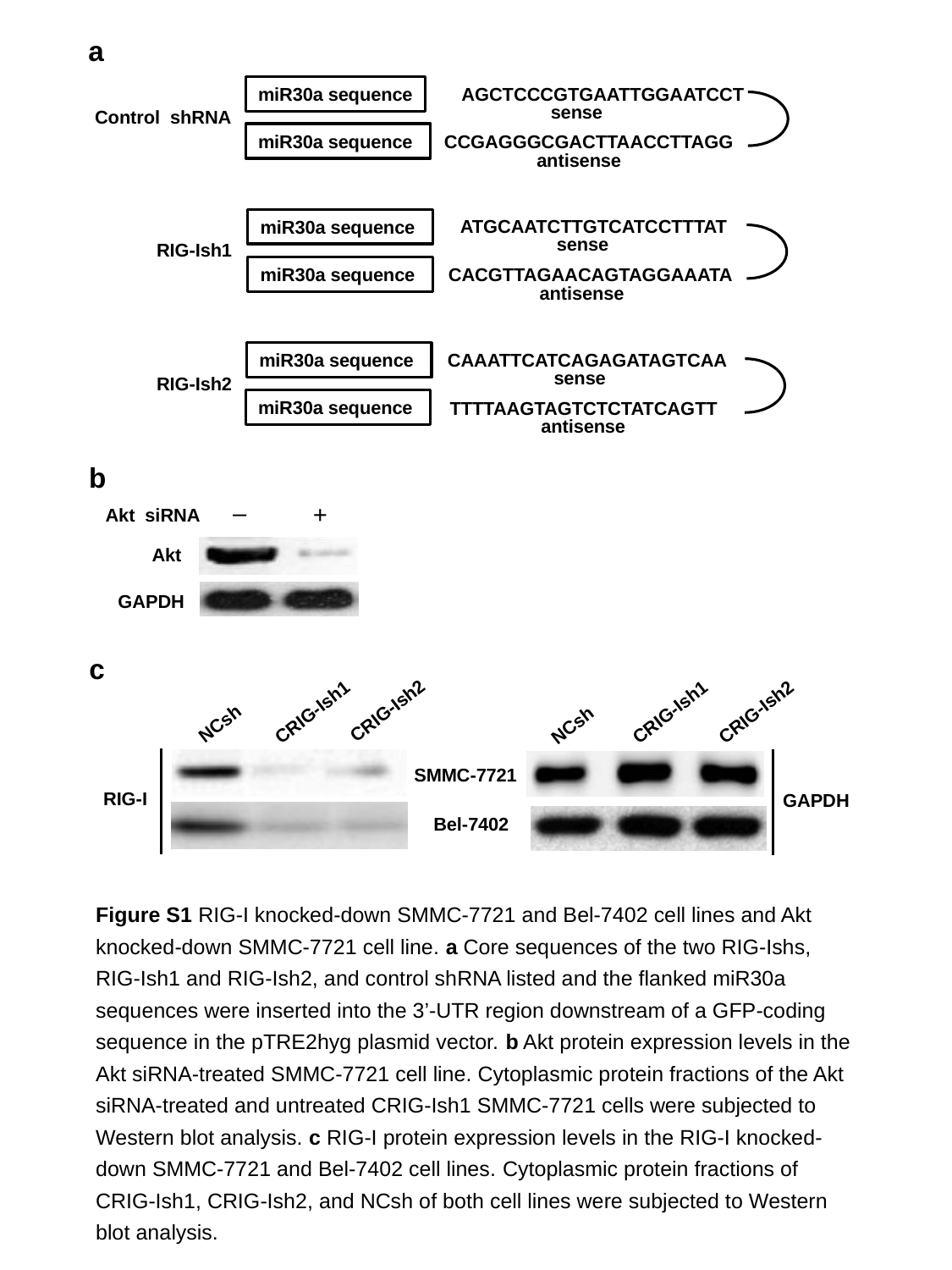

a
miR30a sequence
AGCTCCCGTGAATTGGAATCCT
sense
Control shRNA
miR30a sequence
CCGAGGGCGACTTAACCTTAGG
antisense
ATGCAATCTTGTCATCCTTTAT
miR30a sequence
sense
RIG-Ish1
CACGTTAGAACAGTAGGAAATA
miR30a sequence
antisense
CAAATTCATCAGAGATAGTCAA
miR30a sequence
sense
RIG-Ish2
miR30a sequence
TTTTAAGTAGTCTCTATCAGTT
antisense
b
–
+
Akt siRNA
Akt
GAPDH
c
CRIG-Ish1
CRIG-Ish2
CRIG-Ish1
 CRIG-Ish2
NCsh
NCsh
SMMC-7721
RIG-I
GAPDH
Bel-7402
Figure S1 RIG-I knocked-down SMMC-7721 and Bel-7402 cell lines and Akt knocked-down SMMC-7721 cell line. a Core sequences of the two RIG-Ishs, RIG-Ish1 and RIG-Ish2, and control shRNA listed and the flanked miR30a sequences were inserted into the 3’-UTR region downstream of a GFP-coding sequence in the pTRE2hyg plasmid vector. b Akt protein expression levels in the Akt siRNA-treated SMMC-7721 cell line. Cytoplasmic protein fractions of the Akt siRNA-treated and untreated CRIG-Ish1 SMMC-7721 cells were subjected to Western blot analysis. c RIG-I protein expression levels in the RIG-I knocked-down SMMC-7721 and Bel-7402 cell lines. Cytoplasmic protein fractions of CRIG-Ish1, CRIG-Ish2, and NCsh of both cell lines were subjected to Western blot analysis.
