# Supplementary material for: Induction of tolerogenic dendritic cells by activated TGF-β/Akt/Smad2 signaling in RIG-I-deficient stemness-high human liver cancer cells
Source: BMC Cancer. 2019 May 14;19:439. doi: 10.1186/s12885-019-5670-9 (PMC6515680; doi:10.1186/s12885-019-5670-9)
Supplement: Supplementary file 2 — Table S1. The primer sequences for quantitative real-time PCR used in the study. (DOCX 14 kb) [file 12885_2019_5670_MOESM2_ESM.docx]

**Table S1** The primer sequences for quantitative real-time PCR used in the study

| Gene | Sequence |
| --- | --- |
| GAPDH | Forward: 5’-ACGGGAAGCTCACTGGCATGG-3’ |
|  | Reverse: 5’-GGTCCACCACCCTGTTGCTGTA-3’ |
| Sox2 | Forward: 5’-CGAGTGGAAACTTTTGTCGGA-3’ |
|  | Reverse: 5’-TGTGCAGCGCTCGCAG-3’ |
| Oct3/4 | Forward: 5’-GTGGAGAGCAACTCCGATG-3’ |
|  | Reverse: 5’-TGCTCCAGCTTCTCCTTCTC-3’ |
| Nanog | Forward: 5’-ACCAGTCCCAAAGGCAAACA -3’ |
|  | Reverse: 5’- TCTGCTGGAGGCTGAGGTAT -3’ |
| c-Myc | Forward: 5’-GGAACGAGCTAAAACGGAGCT-3’ |
|  | Reverse: 5’-GGCCTTTTCATTGTTTTCCAACT-3’ |
| β-catenin | Forward: 5’-TCGAAGCATGGACCAAGACC-3’ |
|  | Reverse: 5’-GGCGATGGGAATGTCATTGC-3’ |
| Klf4 | Forward: 5’-CCGCTCCATTACCAAGAGCT-3’ |
|  | Reverse: 5’-ATCGTCTTCCCCTCTTTGGC-3’ |
| IL-6 | Forward: 5’-TTCGGTCCAGTTGCCTTCTC -3’ |
|  | Reverse: 5’-AGCTGCGCAGAATGAGATGA-3’ |
| IL-12 | Forward: 5’-AGTTTGGCCAGAAACCTCCC-3’ |
|  | Reverse: 5’-TCCGGTTCTTCAAGGGAGGA-3’ |
| IL-10 | Forward: 5’-AGCTCAGCACTGCTCTGTTG-3’ |
|  | Reverse: 5’-TCGTATCTTCATTGTCATGTAGGCT-3’ |
| PTGES2 | Forward: 5’-GCCCAGCAAGTGTATGGTG-3’ |
|  | Reverse: 5’-GTACACATTGGGGGAGATCAG-3’ |
| VEGF | Forward: 5'-CACATAGGAGAGATGAGCT-3' |
|  | Reverse: 5'-CCGCCTCGGCTTGTCACAT-3' |
| TGF-β1 | Forward: 5'-CCCCTACATTTGGAGCCTGG-3' |
|  | Reverse: 5'- GCACGATCATGTTGGACAGC-3' |
