# Supplementary material for: Induction of tolerogenic dendritic cells by activated TGF-β/Akt/Smad2 signaling in RIG-I-deficient stemness-high human liver cancer cells
Source: BMC Cancer. 2019 May 14;19:439. doi: 10.1186/s12885-019-5670-9 (PMC6515680; doi:10.1186/s12885-019-5670-9)

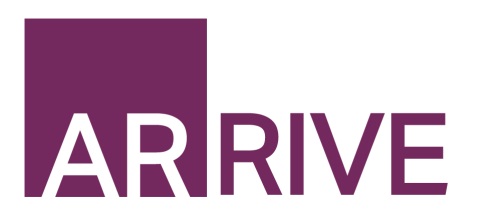


The ARRIVE Guidelines Checklist

Animal Research: Reporting In Vivo Experiments

Carol Kilkenny^1^, William J Browne^2^, Innes C Cuthill^3^, Michael Emerson^4^ and Douglas G Altman^5^

*^1^The National Centre for the Replacement, Refinement and Reduction of Animals in Research, London, UK, ^2^School of Veterinary Science, University of Bristol, Bristol, UK, ^3^School of Biological Sciences, University of Bristol, Bristol, UK, ^4^National Heart and Lung Institute, Imperial College London, UK, ^5^Centre for Statistics in Medicine, University of Oxford, Oxford, UK.*

|  | | ITEM | RECOMMENDATION | Section/ Paragraph |
| --- | --- | --- | --- | --- |
| 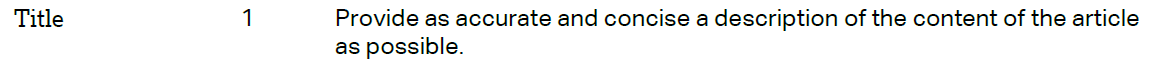 | | | Title |  |
| 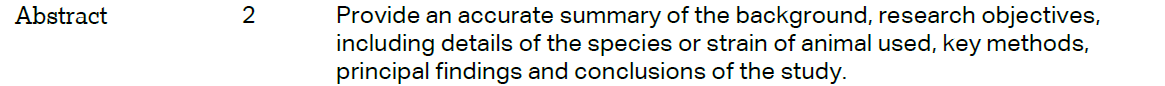 | | | Abstract |  |
| INTRODUCTION | | |  |  |
| 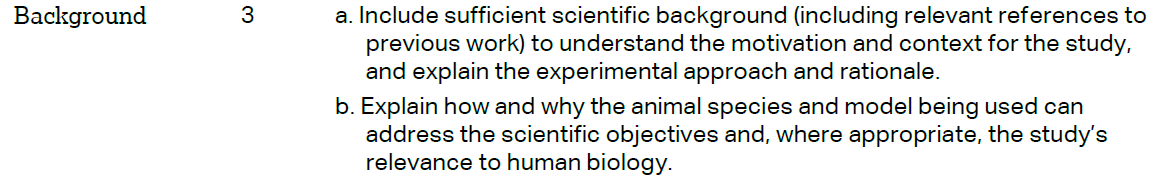 | | | Paragraphs 1-3  Paragraph 3 |  |
| 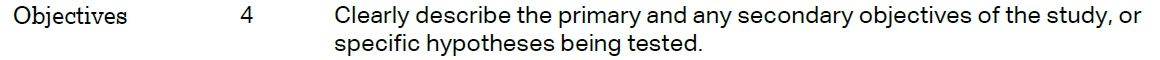 | | | Paragraph 4 |  |
| METHODS | | |  |  |
| 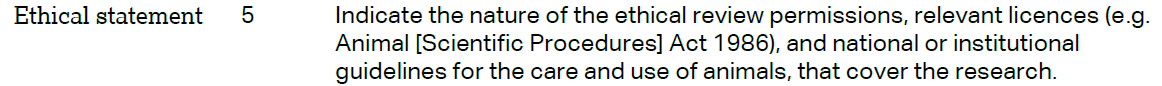 | | | Subsection 11 |  |
| 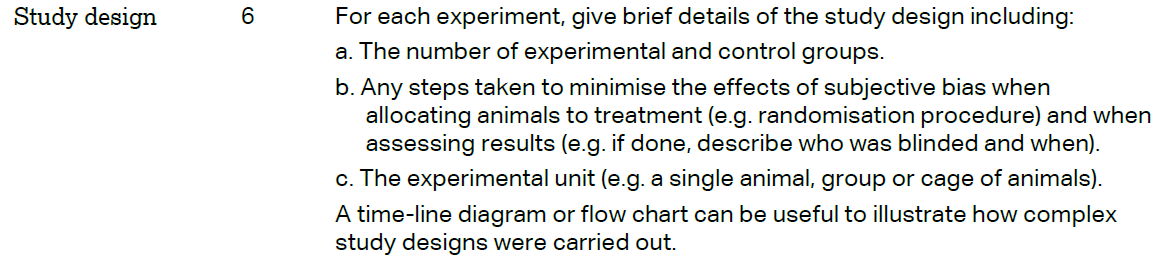 | | | Subsection 11 |  |
| 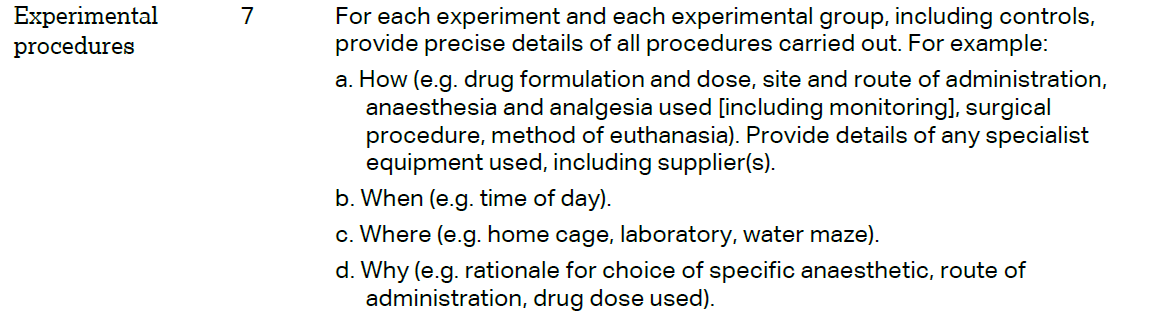 | | | Subsection 11 |  |
| 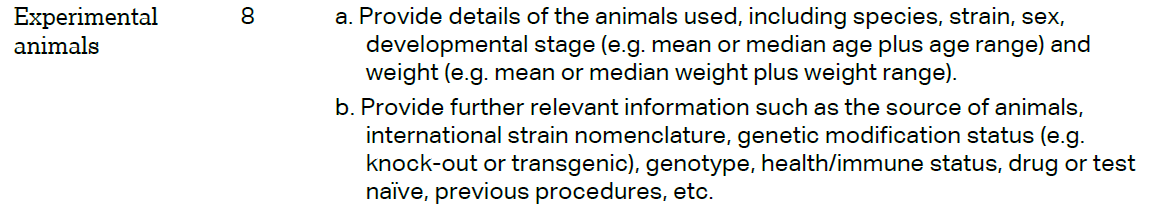 | | | Subsection 11  Results  Subsection 5 |  |

The ARRIVE guidelines. Originally published in *PLoS Biology*, June 2010^1^

| 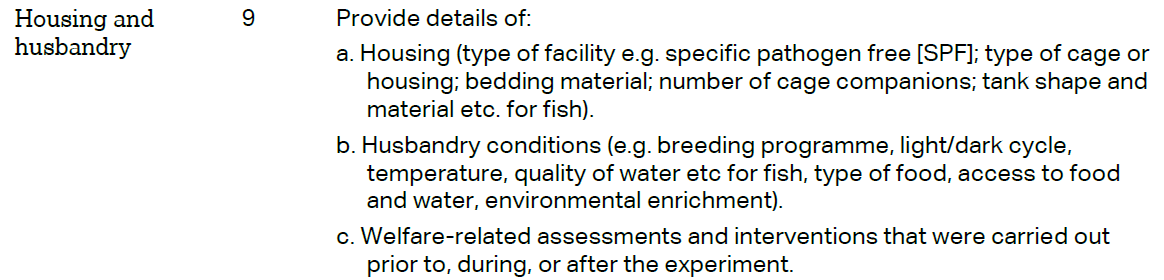 | Subsection 11 | |
| --- | --- | --- |
| 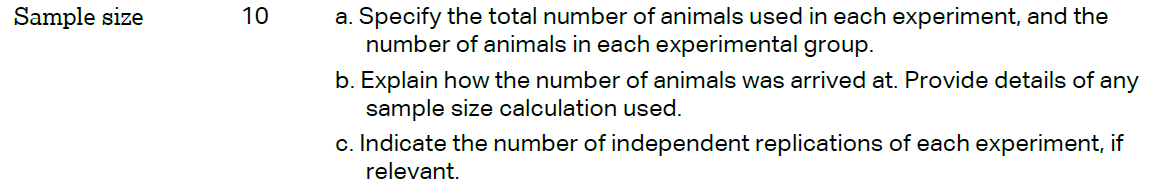 | Subsection 11 | |
| 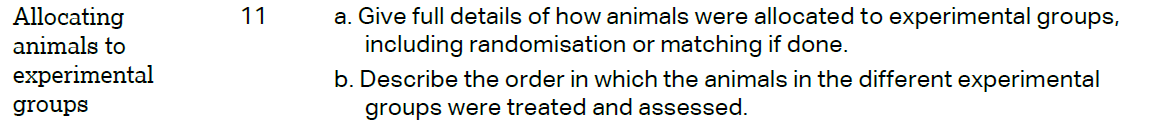 | Subsection 11 | |
| 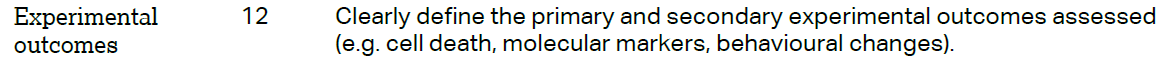 | Subsection 11 | |
| 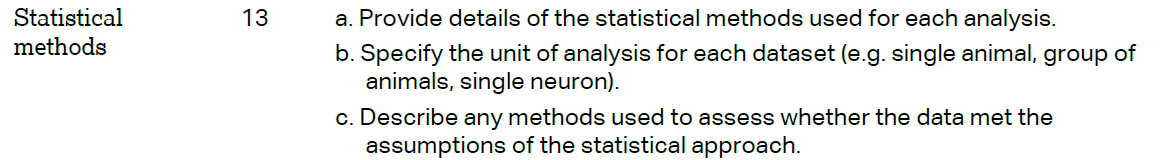 | Subsection 13 | |
| RESULTS |  | |
| 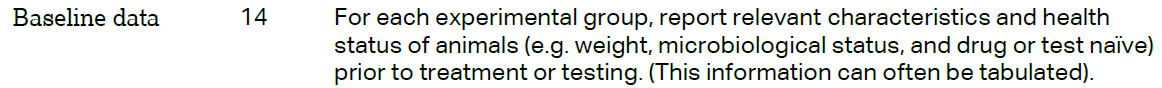 | Subsection 5 | |
| 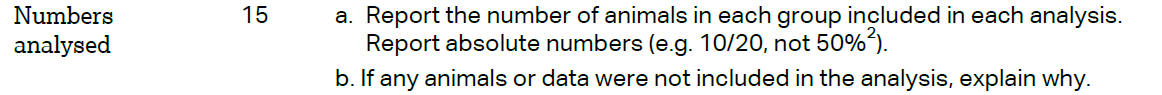 | Subsection 5 | |
| 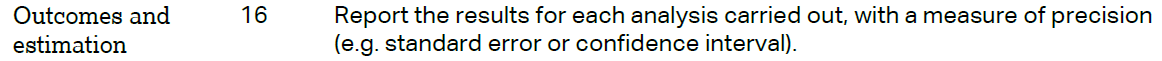 | Subsection 5  Figure 5 | |
| 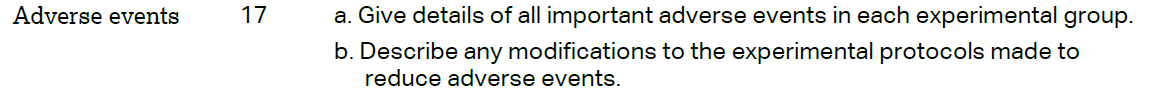 | Subsection 5 | |
| DISCUSSION |  | |
| 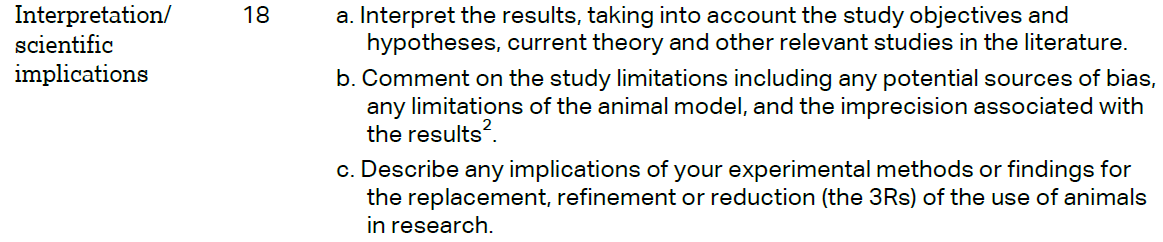 | Throughout  Paragraph 1  Paragraph 1 | |
| 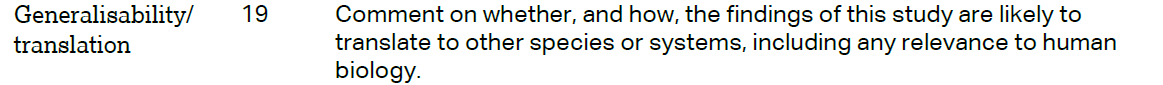 | Paragraph 1 | |
| 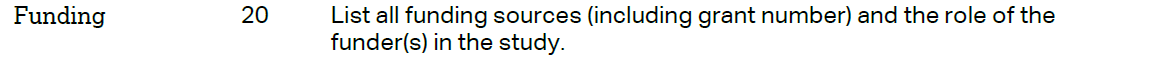 | | Declara-tions  Funding |


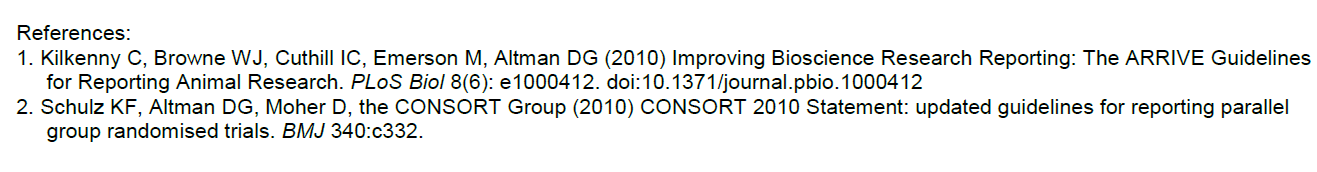

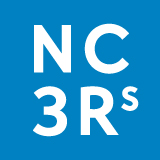

Supplement: Supplementary file 3 — Checklist S1. NC3Rs ARRIVE Guideline Checklist (MZ et al.). (DOCX 658 kb) [file 12885_2019_5670_MOESM3_ESM.docx]
